# The role of lumbar core muscle morphology in residual chronic low back pain after PLIF: a retrospective analysis

**DOI:** 10.3389/fsurg.2026.1833183

**Published:** 2026-07-01

**Authors:** Jiajia Xu, Ning Xiao, Guangwei Liu, Zhihuang Sun, Zhuo Wang, Xianyu Zhang, Jian Jiang, Guobao Wu

**Affiliations:** Department of Orthopedics, ShangRao People’s Hospital, Nanchang University, Shangrao, Jiangxi, China

**Keywords:** cross-sectional area, lumbar muscles, posterior lumbar interbody fusion, quantitative computed tomography, residual chronic low back pain

## Abstract

**Background:**

The purpose of this study was to analyze the correlation between the cross-sectional area (CSA) and density of lumbar core muscles and residual chronic low back pain (rCLBP) after Posterior Lumbar Interbody Fusion (PLIF).

**Methods:**

In this retrospective study, patients who underwent PLIF surgery were stratified into two groups based on postoperative pain status: those with rCLBP and a non-pain control group (NrCLBP). Preoperative lumbar computed tomography (CT) measured the CSA and mean density (HU) of bilateral psoas major, quadratus lumborum, and posterior paraspinal muscles. Preoperative confounding factors, including Cobb angle, endplate injury, Modic changes, and Pfirrmann grade, were also included. Logistic regression analysis was used to identify independent risk factors for rCLBP.

**Results:**

This study ultimately included 46 rCLBP patients and 51 NrCLBP patients. Univariate analysis revealed that longer disease duration and larger Cobb angle were associated with a higher likelihood of rCLBP. However, this difference was not statistically significant in multivariate analysis. Multivariate logistic regression analysis revealed that lower CSA of the psoas major (OR = 1.215, *P* < 0.05) and paraspinal muscles (OR = 1.159, *P* < 0.05), and lower mean HU of the quadratus lumborum (OR = 1.197, *P* < 0.05) were independently associated with rCLBP.

**Conclusion:**

Preoperatively reduced CSA of the psoas major and paraspinal muscles independently predicted increased risk of rCLBP. When assessing the impact of the quadratus lumborum, muscle density may serve as a more sensitive imaging biomarker. However, causality cannot be inferred from this retrospective design, and postoperative muscle recovery remains unassessed with preoperative CT-only data.

## Introduction

Posterior Lumbar Interbody Fusion (PLIF) is one of the most widely used lumbar interbody fusion techniques in clinical practice, primarily employed to treat lumbar degenerative diseases with segmental instability (1). With the development of pedicle screws and interbody fusion implants, PLIF offers the benefits of immediate postoperative biomechanical stability and a high fusion rate (2). Although PLIF successfully addresses primary issues such as neural compression and spinal instability, 30.3% of patients may still experience persistent discomfort, including residual chronic low back pain (rCLBP, pain ≥3 months postoperatively with visual analogue scale ≥4) (3, 4), significantly impacting quality of life, functional recovery, and healthcare costs. Analgesic therapies such as intramuscular and intrathecal trigger point injections with lidocaine have not yielded long-term benefits (5). The mechanisms underlying rCLBP after PLIF remain incompletely elucidated, and clinical treatment options are limited. Further exploration of its etiology holds significant clinical value.

Traditionally, rCLBP has been attributed to factors such as tissue damage caused by PLIF surgery, intraoperative traction on the dural sac and nerve roots, adjacent segment degeneration, and compensatory stress changes resulting from the stiffness of the fused segments (6, 7). However, clinically, we observe that some patients exhibit no signs of tissue inflammation or edema, no adjacent segment disease (ASD), and no solid bony fusion on postoperative imaging, yet pain persists. Therefore, some scholars suggest that rCLBP may be related to psychological factors such as postoperative anxiety and pain sensitization (8). Angadi et al. (9) found that patients with higher preoperative pain sensitivity had higher pain scores and greater analgesic requirements at any point after lumbar fusion surgery. Sun et al. (10) further discovered that 46.5% of PLIF patients experience low back pain in the short term (<7 days). In addition to the degree of depression, the cross-sectional area (CSA) of the psoas and multifidus muscles is also an independent risk factor. Here, it is critical to note that muscle CSA, as measured by computed tomography (CT), serves as a key indicator of muscle quantity and is strongly associated with strength reserve. Conversely, muscle density, quantified in Hounsfield Units (HU) on CT, directly reflects muscle quality, with lower HU values indicating increased myosteatosis. Importantly, myosteatosis is increasingly recognized not merely as a marker of muscle degeneration, but also as a contributor to a pro-inflammatory environment and neural sensitization, both potent drivers of rCLBP. This suggests that non-osseous structures (e.g., changes in low back muscle morphology) may also be significant drivers of pain. However, most current studies focus primarily on bony fusion or neural decompression outcomes, lacking systematic assessment of the muscular system.

Song et al. (11) followed 163 patients who underwent minimally invasive lumbar discectomy for an average of 16.5 months and found that patients with poorer preoperative lumbar muscle health experienced longer postoperative functional recovery times. After PLIF, back muscle strength may decline within the first 3 months postoperatively. Patients who regularly perform back muscle exercises show significant improvement in back strength, reduced pain, and decreased disability by 12 months postoperatively (12). Previous limited studies on postoperative muscle morphology were confined to short-term follow-up (e.g., within 3 months) and unable to explain the correlation between rCLBP and lumbar muscle morphology. This study aimed to investigate whether preoperative lumbar muscle morphology—specifically, the cross-sectional area and density of the psoas major, quadratus lumborum, and paraspinal muscles—is associated with the development of rCLBP following PLIF surgery. We hypothesized that patients with lower muscle CSA and density would be more likely to develop rCLBP after PLIF.

## Materials and methods

### Patient characteristics

This study included patients who underwent PLIF surgery in our department between January 2020 and December 2023. We retrospectively analyzed the correlation between lumbar muscle morphology and postoperative rCLBP. Patients with persistent low back pain lasting more than 3 months after PLIF surgery and a Visual Analogue Scale (VAS, 0–10) score ≥4 were classified into the rCLBP group; the remaining patients were assigned to the no residual pain group (NrCLBP). Inclusion criteria: (1) Age 45–80 years, this interval aligns with the typical demographic profile of patients undergoing PLIF surgery. Older patients (beyond 80 years) often exhibit greater physiological decline and more comorbidities, while younger patients (under 45) generally maintain higher daily activity levels. Both extremes could introduce variability that compromises the comparability of results; (2) Body Mass Index (BMI) 18.5–25 kg/m^2^ (to minimize confounding effects from conditions such as extreme underweight or obesity); (3) Diagnosed with degenerative lumbar spinal stenosis and undergoing primary PLIF surgery, and flexion-extension radiographs at 3 months post-operation demonstrated solid fusion at the surgical level(s); (4) Complete clinical follow-up and imaging data. Exclusion criteria: (1) History of prior lumbar surgery; (2) Comorbidities including neuromuscular disorders, severe osteoporosis, malignant tumors, metabolic diseases severely affecting endocrine function or psychiatric disorders; (3) History of lumbar trauma, fractures, infections, or steroid injections; (4) Use of medications affecting bone metabolism or muscle mass (e.g., glucocorticoids, thyroid hormones); (5) PLIF surgical failure with surgery-related complications (excluding pain), such as implant loosening, malpositioned implants, secondary vertebral fractures, postoperative infection, or poor wound healing. All surgeries were performed jointly by the same attending physician and chief physician. This study was approved by the hospital's Ethics Committee (IRB.2024-148). In accordance with ethical requirements, patient information was anonymized and informed consent was obtained from all participants.

### Quantitative assessment of lumbar muscles

**Figure 1:**
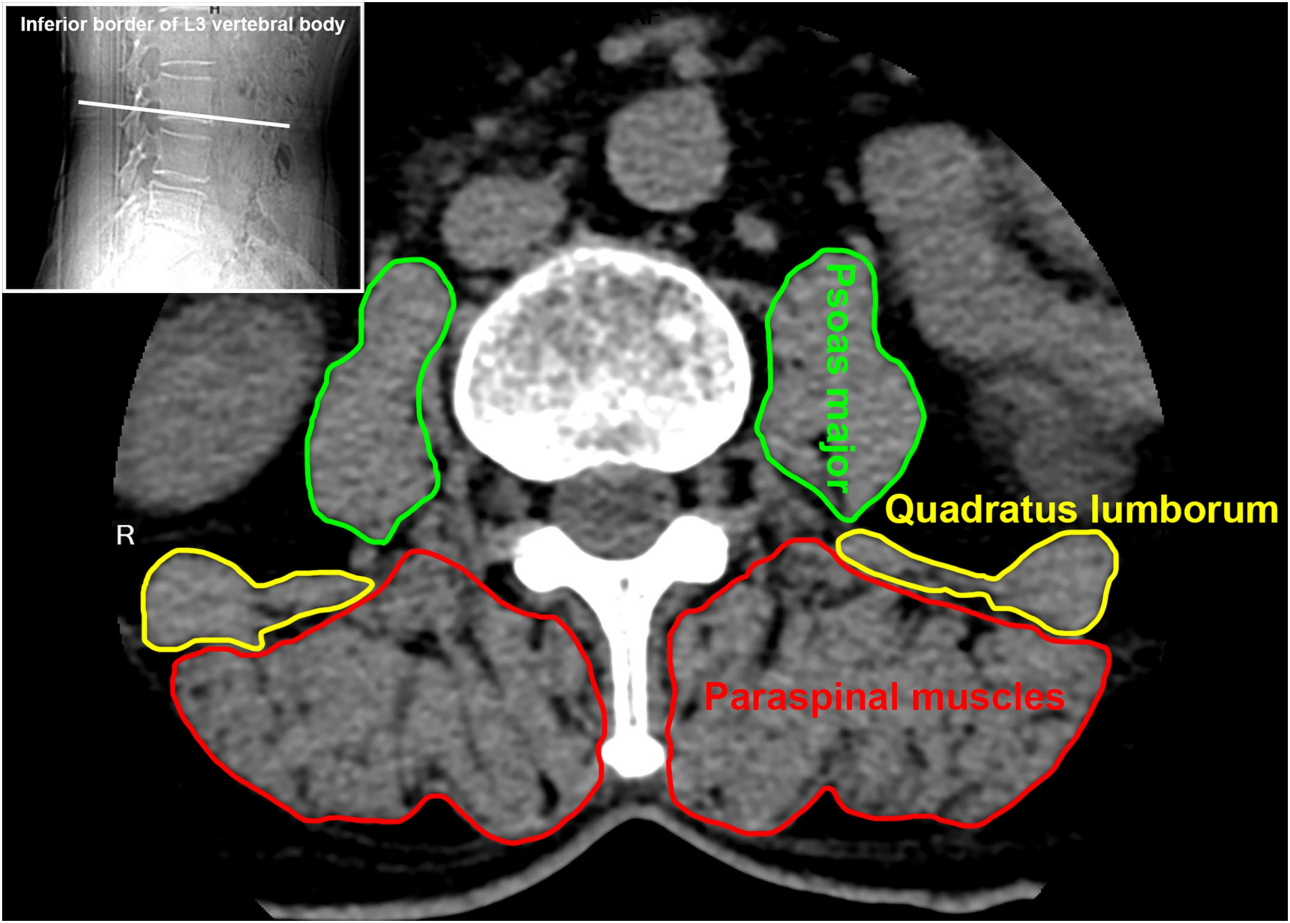
Schematic diagram of lumbar muscle morphology measurement. At the level of the lower endplate of the L3 vertebra on Computed Tomography (CT), the cross-sectional area (CSA, cm^2^) and mean density (Hounsfield Units, HU) of the bilateral Psoas major (green), Quadratus lumborum (yellow), and Paraspinal muscles (red) were measured.

Lumbar muscle parameters were measured using preoperative lumbar CT scans (acquired at 120 kV, covering vertebrae L3–S1, with a slice thickness of 3.75 mm and increment of 1.25 mm). At the level of the inferior endplate of the L3 vertebra, two experienced doctors jointly delineated the bilateral psoas major, quadratus lumborum, and paraspinal muscles (erector spinae and multifidus) on the Picture Archiving and Communication Systems (PACS) workstation using the manual ROI tool. The CSA (cm^2^) and mean density (HU) were automatically computed by the software (Figure 1). The L3 vertebra typically lies at the mid-lumbar region, where the psoas major muscle exhibits its fullest and most uniform cross-sectional morphology. This anatomical consistency allows for precise and reproducible delineation of muscle boundaries, minimizing measurement variability. Thus, the L3 level offers an optimal balance between anatomical centrality, morphological representativeness, clinical feasibility, and standardization across studies (13–15). The Cobb angle was measured using preoperative spinal radiographs. Preoperative lumbar Magnetic Resonance Imaging (MRI) was used to evaluate superior adjacent vertebral endplate injuries, Modic changes, and the Pfirrmann grade of superior adjacent segment discs (16).

### Statistical analysis

Data were analyzed using IBM SPSS Statistics V20 software (IBM Corp., USA), considering a significance threshold of *P* < 0.05. Normally distributed data are presented as mean ± standard deviation; non-normally distributed data are presented as median ± standard error. Intergroup comparisons were performed using non-parametric tests or chi-square tests. Logistic regression analysis was used to assess the correlation between rCLBP and the following variables: CSA and mean density of each muscle group, age, BMI, sex, preoperative disease duration, Cobb angle, surgical segments (single/multiple), superior adjacent vertebral endplate injuries, Modic changes, and Pfirrmann grade (no multicollinearity: VIF < 5, Tolerance > 0.2). For logistic regression analysis, univariate analysis was first performed, followed by multivariate analysis incorporating variables with *P* < 0.10. Additionally, linear regression analysis was used to examine the linear relationships between the CSA of different muscle groups and between CSA and mean density.

## Results

**Figure 2:**
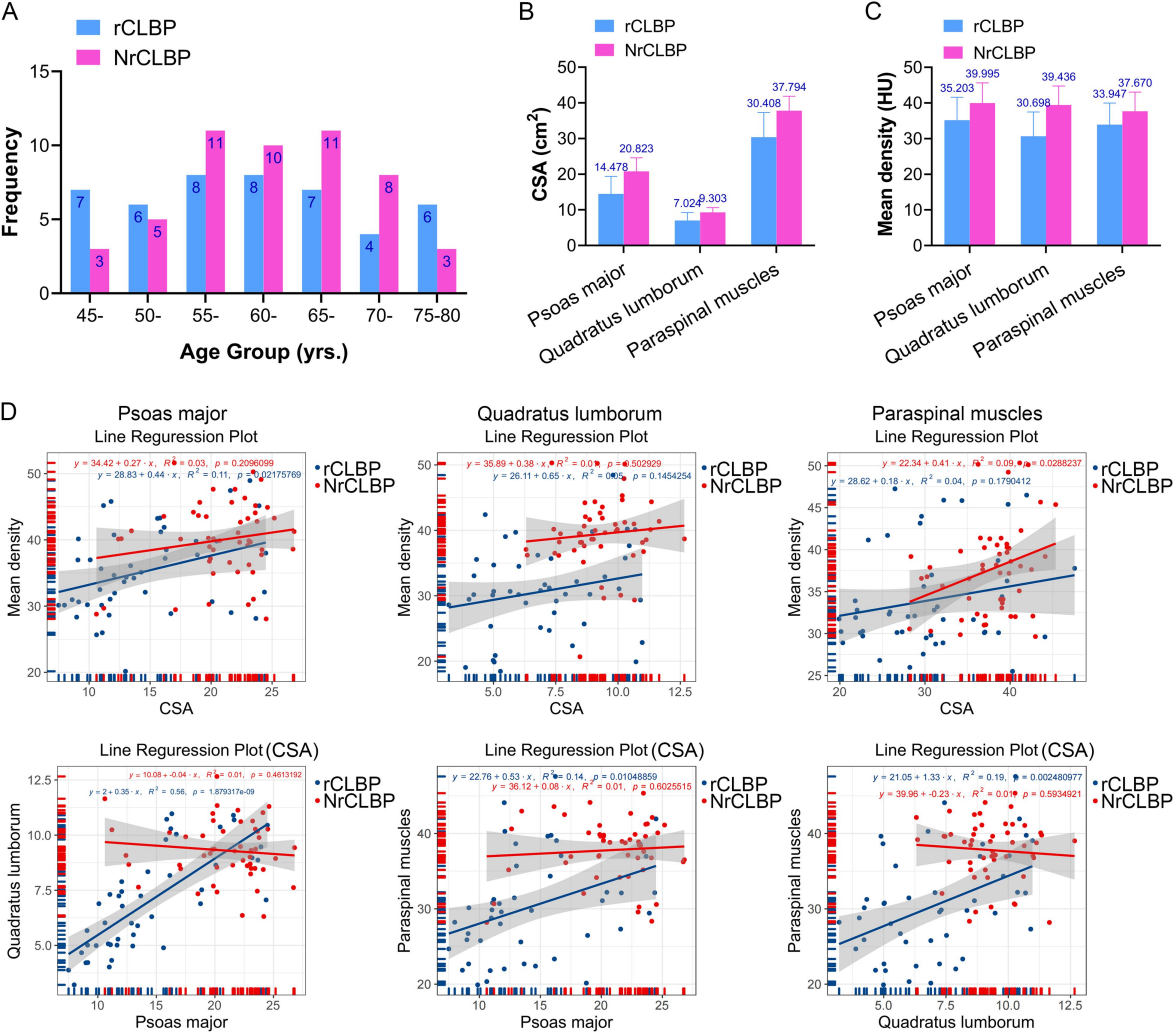
Linear correlation analysis of muscle cross-sectional area (CSA) and mean density (HU). **(A)** Age frequency distribution of patients in the Residual Chronic Low Back Pain (rCLBP) group and the No Residual Pain (NrCLBP) group. **(B)** CSA of Psoas major, Quadratus lumborum, and Paraspinal muscles (Mean ± Standard Deviation). **(C)** HU of Psoas major, Quadratus lumborum, and Paraspinal muscles (Mean ± Standard Deviation). **(D)** Linear regression analysis between the CSA and HU of the Psoas major, Quadratus lumborum, and Paraspinal muscles, or between the CSA of individual muscles.

This study included 46 patients (Han Chinese ethnicity) with rCLBP (26 females, 20 males) and 51 patients with NrCLBP (23 females, 28 males) (Table 1, age distribution shown in Figure 2A). There was no statistically significant difference in disease duration between the rCLBP group (median 4.500 ± 1.126 years) and the NrCLBP group (median 3.500 ± 0.396 years) (*P* = 0.226, Independent-Samples Mann–Whitney *U* Test). However, the preoperative Cobb angle was slightly larger in the rCLBP group (9.620 ± 3.884) than in the NrCLBP group (7.829 ± 3.141) (*P* = 0.020, Independent-Samples Mann–Whitney *U* Test). For surgical segments (single/multiple, *P* = 0.143, Pearson Chi-Square test), endplate injury in the adjacent upper vertebrae (*P* = 0.094, Pearson Chi-Square test), Modic changes (*P* = 0.057, Pearson Chi-Square test), and Pfirrmann grade (*P* = 0.650, Cochran–Mantel–Haenszel test), there were no statistically significant differences between the rCLBP and NrCLBP groups (*P* > 0.05).

**Table 1:** Patient demographics (*n* = 97).

Variables		*N* (*n* %) or M ± S*/**	*P* value, rCLBP vs. NrCLBP
Gender (*N*)	Male	48 (49.500)	
	Female	49 (50.500)	
BMI (kg/m^2^)		21.907 ± 1.920*	
rCLBP group^a^		46 (47.400)	
NrCLBP group^a^		51 (52.600)	
Duration of illness (years)	rCLBP	4.500 ± 1.126**	0.226
	NrCLBP	3.500 ± 0.396**	
Preop-Cobb's angle (°)	rCLBP	9.620 ± 3.884*	0.020
	NrCLBP	7.829 ± 3.141*	
Operative segments	Single, rCLBP	23 (23.711)	0.143
	Single, NrCLBP	33 (34.021)	
	Multiple, rCLBP	23 (23.711)	
	Multiple, NrCLBP	18 (18.557)	
Preop-endplate injury in the adjacent upper vertebrae	Yes, rCLBP	24 (24.742)	0.094
	Yes, NrCLBP		
	No, rCLBP	22 (22.680)	
	No, NrCLBP		
Preop-Modic changes in the adjacent upper vertebrae	Yes, rCLBP	17 (17.526)	0.057
	Yes, NrCLBP		
	No, rCLBP	29 (29.897)	
	No, NrCLBP		
Preop-Pfirrmann classification of the adjacent upper segment, rCLBP	I	8 (8.247)	0.650
	II	15 (15.464)	
	III	10 (10.309)	
	IV	10 (10.309)	
	V	3 (3.093)	
Preop-Pfirrmann classification of the adjacent upper segment, NrCLBP	I	12 (12.371)	
	II	16 (16.495)	
	III	14 (14.433)	
	IV	8 (8.247)	
	V	1 (1.031)	

*/**M ± S: *mean ± standard deviation or **median ± standard error.

a rCLBP: persistent low back pain lasting more than 3 months after PLIF surgery and a visual analogue scale ≥4; NrCLBP, no residual pain group.

Figures 2B,C display the CSA and mean density of the Psoas major (CSA: rCLBP/NrCLBP = 14.478/20.823), Quadratus lumborum (CSA: rCLBP/NrCLBP = 7.024/9.303), and Paraspinal muscles (CSA: rCLBP/NrCLBP = 30.408/37.794) for the rCLBP and NrCLBP groups, respectively. Linear regression models revealed no significant linear relationship between CSA and mean density, nor between the CSAs of the different muscle groups (Figure 2D).

Univariate logistic regression analysis revealed that Disease duration (*P* = 0.043, OR = 0.899, 95% CI = 0.811–0.997), Preop-Cobb's angle (*P* = 0.017, OR = 0.865, 95% CI = 0.768–0.974), Preop-Endplate injury in the adjacent upper vertebrae (*P* = 0.096, OR = 0.500, 95% CI = 0.221–1.130), Preop-Modic changes in the adjacent upper vertebrae (*P* = 0.060, OR = 0.416, 95% CI = 0.167–1.038), and the CSA and mean density of each muscle group might be associated with rCLBP. These risk factors were subsequently included in the multivariate analysis (Table 2, Hosmer–Lemeshow Test *P* = 0.532). Multivariate analysis demonstrated that lower CSA of the Psoas major (*P* = 0.027, OR = 1.215, 95% CI = 1.023–1.444) and Paraspinal muscles (*P* = 0.031, OR = 1.159, 95% CI = 1.014–1.326) and lower mean density of the Quadratus lumborum (*P* = 0.002, OR = 1.197, 95% CI = 1.066–1.344) were associated with a higher risk of rCLBP (*P* < 0.05).

**Table 2:** Risk factors for residual back pain after lumbar spine surgery identified by logistic regression analysis.

		Univariate analysis			Multivariate analysis			Collinearity statistics	
Factors		OR	95% CI for OR	*P*	OR	95% CI	*P*	Tolerance	VIF
Age group (by 5 years)	45∼	0.857	0.124–5.944	0.876					
	50∼	1.667	0.269–10.334	0.583					
	55∼	2.750	0.524–14.439	0.232					
	60∼	2.500	0.471–13.265	0.282					
	65∼	3.143	0.586–16.845	0.181					
	70∼	4.000	0.639–25.020	0.138					
	75∼	-	-	-					
Body mass index	(kg/m^2^)	0.916	0.742–1.130	0.410					
Gender	(female/male)	0.632	0.283–1.410	0.262					
Duration of illness	(years)	0.899	0.811–0.997	0.043*	0.974	0.846–1.122	0.715	0.641	1.561
Preop-Cobb's angle	(°)	0.865	0.768–0.974	0.017*	0.997	0.801–1.242	0.981	0.792	1.262
Operative segments	(single/multiple)	1.833	0.812–4.141	0.145					
Preop-endplate injury in the adjacent upper vertebrae	(Yes/No)	0.500	0.221–1.130	0.096*	1.504	0.177–12.777	0.709	0.407	2.457
Preop-Modic changes in the adjacent upper vertebrae	(Yes/No)	0.416	0.167–1.038	0.060*	0.508	0.054–4.739	0.552	0.400	2.503
Preop-Pfirrmann classification of the adjacent upper segment		0.813	0.569–1.162	0.257					
CSA of psoas major bilaterally	(cm^2^)	1.331	1.193–1.485	0.000*	1.215	1.023–1.444	0.027**	0.398	2.511
Mean density of psoas major bilaterally	(HU)	1.140	1.059–1.228	0.001*	1.037	0.912–1.179	0.579	0.649	1.540
CSA of quadratus lumborum bilaterally	(cm^2^)	1.905	1.445–2.513	0.000*	1.316	0.880–1.968	0.181	0.484	2.066
Mean density of quadratus lumborum bilaterally	(HU)	1.275	1.154–1.409	0.000*	1.197	1.066–1.344	0.002**	0.645	1.550
CSA of paraspinal muscles bilaterally	(cm^2^)	1.246	1.138–1.364	0.000*	1.159	1.014–1.326	0.031**	0.575	1.738
Mean density of paraspinal muscles bilaterally	(HU)	1.125	1.040–1.216	0.003*	0.953	0.834–1.089	0.480	0.636	1.571

-, Reference.

* Risk factors with *P* < 0.10 in univariate analysis were included in a multivariate analysis.

** Independent risk factors (*P* < 0.05) was identified by a multivariate logistic regression analysis.

## Discussion

This study retrospectively analyzed preoperative lumbar CT data from 97 patients undergoing PLIF surgery to systematically evaluate the relationship between morphological indicators (CSA and mean density) of the core lumbar muscles (Psoas major, Quadratus lumborum, Paraspinal muscles) and the occurrence of rCLBP postoperatively. The core findings are that smaller CSA of the Psoas major and paraspinal muscles, along with lower mean density of the Quadratus lumborum, are independent risk factors for developing rCLBP after PLIF. Multivariable analysis revealed that a smaller preoperative CSA of the psoas major (OR = 1.215, 95% CI = 1.023–1.444) and paraspinal muscles (OR = 1.159, 95% CI = 1.014–1.326), alongside a lower quadratus lumborum density (OR = 1.197, 95% CI = 1.066–1.344), were significant independent risk factors for rCLBP. This result highlights the potential value of preoperative lumbar muscle morphological characteristics in predicting rCLBP, providing new insights into the pathophysiological mechanisms of rCLBP and informing the development of preventive strategies.

While this study focused on muscle morphology, we recognize that factors like age, sex, and number of fusion levels could plausibly modify the association between muscle health and rCLBP. Our subgroup analyses did not reveal significant effect modifications by these variables within our cohort, though the sample size limited the power for such interactions. Future larger studies are warranted to definitively explore these potential relationships.

Clinically, rCLBP may be influenced by multiple factors, including surgical, biomechanical, and psychosocial factors. This study confirmed that decreased CSA of the Psoas major and Paraspinal muscles (erector spinae, multifidus) are independent predictors of rCLBP occurrence. This finding aligns with the results of Sun et al. (10), who reported a correlation between the areas of the psoas major and multifidus muscles and short-term pain after PLIF, extending this association into the chronic phase (>3 months). From a biomechanical perspective, the psoas major is an important hip flexor and lumbar stabilizer. These findings resonate with the conceptual model of spinal stability proposed by Panjabi (17), where the musculoskeletal subsystem (comprising muscles, tendons, and ligaments) is essential for maintaining spinal equilibrium and preventing injury. Paraspinal muscles, particularly the deep multifidus, are key segmental stabilizers, providing posterior tension band support and resistance to shear forces. While PLIF reconstructs spinal bony stability, the rigidity of the fused segments inevitably increases the biomechanical burden on adjacent segments and alters the overall pattern of spinal load transmission (18). Pre-existing atrophy of these muscles signifies insufficient muscle strength reserve, a core component of sarcopenia as defined by the European Working Group on Sarcopenia in Older People (EWGSOP) (19, 20). Postoperatively, these weakened muscles are less able to effectively compensate for the altered movement patterns and increased stress caused by fusion, making them prone to fatigue, microtrauma, and local inflammatory reactions, which can potentially lead to abnormal loading of facet joints, discs, or ligaments, ultimately manifesting as rCLBP (21). Furthermore, muscle atrophy is often accompanied by changes in muscle fiber type (e.g., reduction in type II fast-twitch fibers) and abnormal innervation, further diminishing the muscles' rapid response and stabilization capacity (22). This emphasis on preoperative muscle status as a predictor of postoperative pain is further supported by a recent study by Hu et al. (23), which identified similar risk factors, including muscle-related parameters, for residual low back pain following percutaneous endoscopic lumbar discectomy (PELD). While the surgical approach (PELD vs. PLIF) differs substantially, the consistent finding across procedures that preoperative muscle quality and morphology influence pain outcomes underscores the fundamental role of muscle health in postoperative recovery and highlights its potential as a modifiable risk factor.

A particularly striking and relatively novel finding of this study is that the mean density (HU value) of the Quadratus lumborum, rather than its CSA, is an independent predictor of rCLBP. The lack of association between Quadratus Lumborum CSA and rCLBP, in contrast to its density, may be related to anatomical and methodological factors. This observation underscores the complexity of defining “muscle health” beyond simple cross-sectional area. This deep muscle's boundaries can be challenging to delineate precisely on CT, especially in the presence of severe fatty infiltration, which may reduce the reliability and reproducibility of CSA measurements and potentially obscure volume-related associations. Our approach of measuring multiple muscle groups at the L3 vertebra is supported by CT-based body composition research, which considers this level a reliable standard for assessing overall skeletal muscle mass and quality (24, 25). Furthermore, studies have shown that relying on a single muscle group (e.g., the psoas alone) can be a poor sentinel for the overall status of the lumbar musculature and may underestimate pathology like myosteatosis (26). HU values on CT primarily reflect the physical density of tissues; in muscle tissue, lower HU values are typically associated with a higher degree of fatty infiltration (myosteatosis). This means that the deterioration in Quadratus lumborum quality (increased fatty infiltration), rather than simply a reduction in volume (decreased CSA), is closely related to rCLBP occurrence. The Quadratus lumborum is a key member of the deep core stabilizing muscles, primarily functioning in lateral flexion of the lumbar spine, stabilization of the 12th rib, and contributing to respiration and pelvic stability. Fatty infiltration not only directly weakens muscle contractile force and endurance but may also cause pain through various mechanisms (27). Goubert et al. (28) previously found higher degrees of lumbar muscle fatty infiltration in patients with persistent chronic LBP compared to those with rCLBP without significant muscle atrophy. Intramuscular fatty infiltration may create a local low-grade inflammatory environment, stimulating nociceptors and leading to impaired motor control and postural stability, thereby increasing the risk of abnormal stress on spinal structures (29). The results of this study suggest that muscle quality (degree of fatty infiltration) may be a more sensitive indicator than muscle volume when assessing the impact of Quadratus lumborum on rCLBP after PLIF.

Another important observation in this study is the lack of a significant linear correlation between muscle CSA and mean density (Figure 2D), as well as the differing predictive roles of morphological indicators for rCLBP across different muscles (e.g., only density was relevant for Quadratus lumborum). This suggests that muscle atrophy (decreased CSA) and myosteatosis (decreased density) are relatively independent pathological processes, potentially driven by different factors (e.g., neurogenic, disuse, metabolic, age-related). The biomechanical roles of different muscle groups in the spine and the mechanisms by which their pathological changes contribute to pain may vary (30–32). The volume (CSA) of the Psoas major and paraspinal muscles may be more critical for maintaining overall strength and stability. At the same time, the quality (density/fatty infiltration) of the Quadratus lumborum, due to its deep location, rich innervation, and unique stabilizing functions, might make it more sensitive to inflammation and neuro-sensitization-related pain pathways. This finding highlights the importance of considering both morphological and qualitative dimensions when evaluating “lumbar muscle health”.

Furthermore, univariate analysis in this study revealed associations between longer disease duration and larger preoperative Cobb angle with rCLBP; however, these associations disappeared after adjusting for muscle morphological indicators and other factors in the multivariate analysis. This suggests that the impact of disease duration and spinal deformity (Cobb angle) on rCLBP may be primarily mediated through their adverse effects on lumbar muscles (especially the psoas major and paraspinal muscles), such as disuse atrophy, muscle fatigue, and damage caused by postural compensation (33). When muscle status is directly measured and controlled, the direct effects of these factors themselves become insignificant.

While our study identifies statistically significant associations between muscle morphology and rCLBP, the determination of precise thresholds for clinical actionability (e.g., specific cut-off values for CSA or HU that mandate prehabilitation) requires further prospective validation. The effect sizes observed, however, suggest a substantial influence of muscle health on postoperative pain outcomes.

These results strongly support Sun et al.'s (10) findings on the correlation between preoperative back muscle (psoas major, multifidus) morphology and post-PLIF pain, extending them to a broader muscle group (Quadratus lumborum) and rCLBP. More importantly, this study reveals the independent value of Quadratus lumborum muscle density in predicting rCLBP and highlights the differential predictive roles of morphological indicators (cross-sectional area vs. density) for different muscle groups. This aligns with the recent trend in non-surgical low back pain research emphasizing the importance of muscle fatty infiltration. Song et al.'s (11) study on the influence of lumbar muscle health status on postoperative functional recovery time is also consistent with the view that insufficient functional reserve of core muscles leads to rCLBP. By employing rigorous quantitative imaging measurements and multivariate analysis while controlling for several potential confounders (body mass index, gender, and surgical levels), this study enhances the credibility of the findings. It provides new, more refined evidence for the central role of muscle factors in rCLBP. The potential clinical applications of these findings are compelling but currently hypothetical.

Preoperative lumbar CT (often a routine examination) can be effectively utilized to identify patients at high risk for rCLBP (muscle atrophy + Quadratus lumborum fatty infiltration) through quantitative measurement of the CSA of the Psoas major and paraspinal muscles, as well as the density of the Quadratus lumborum. This facilitates thorough preoperative communication, sets realistic expectations, and enables the development of more proactive perioperative management plans. For identified high-risk patients, future research could explore the efficacy of implementing intensive, individualized core muscle prehabilitation before elective surgery (34). Such future training programs should not only focus on increasing muscle volume (resistance training) but also on improving muscle quality (e.g., neuromuscular control training, endurance training), with particular emphasis on Quadratus lumborum training. The goal is to maximize preoperative muscle functional reserve. For patients who have already developed rCLBP with significant muscle degeneration, pain management should integrate muscle factors. Beyond conventional pharmacological and interventional treatments, targeted, high-intensity neuromuscular rehabilitation remains the cornerstone of treatment. Exploring interventions targeting muscle fatty infiltration or inflammation (e.g., specific nutritional strategies and certain medications) represents a promising avenue for future research.

However, this study has certain limitations: (1) The retrospective design carries inherent risks of selection and information bias. Causality between muscle morphological changes and rCLBP cannot be established (whether cause, consequence, or concomitant phenomenon); (2) Only preoperative CT data was used for muscle assessment. The lack of data on postoperative dynamic changes in muscle morphology prevents evaluation of the impact of postoperative muscle recovery on rCLBP; (3) The boundaries of the Quadratus lumborum can sometimes be difficult to precisely delineate on CT, especially with fatty infiltration, which may affect the reproducibility of CSA measurements. This could be one reason why the Quadratus lumborum CSA did not show a significant correlation; (4) CT provides static morphological information and cannot assess dynamic functional indicators, such as muscle activation patterns, strength, or endurance. Future studies incorporating electromyography (EMG) or isokinetic strength testing could provide more comprehensive information; (5) Measurements were taken only at the lower border of L3, which may not fully represent the muscle status of the entire lumbar region. Multi-level or volumetric measurements might be superior. We did not use normalization methods such as skeletal muscle index (SMI) or vertebral body normalization when analyzing muscle cross-sectional area. Although we restricted the BMI range of participants to minimize the confounding effect of body size, absolute CSA values may still be influenced by individual differences in body dimensions. Future studies should consider using normalized muscle area values to further improve the accuracy of the results. (6) A limitation of this study is the lack of *a priori* power analysis for sample size estimation, which was constrained by the availability of eligible participants during the study period. (7) While we utilized CT-based measures of muscle mass and density, which are well-validated research tools, our study did not formally diagnose sarcopenia according to full clinical criteria like EWGSOP, as we lacked functional assessment. Future studies integrating imaging with functional measures could further clarify the relationship between sarcopenia and surgical outcomes. Future prospective cohort studies systematically collecting multi-dimensional data—including muscle morphology (MRI/CT), muscle function (strength, EMG), pain, function, and psychosocial factors—at different time points (e.g., preoperatively, 3, 6, 12 months postoperatively) are warranted to clarify causal relationships and dynamic evolution.

## Conclusion

This study provides important evidence that preoperative morphological characteristics of lumbar muscles, specifically decreased CSA of the Psoas major and paraspinal muscles, and reduced density of the quadratus lumborum, are independent risk factors for developing rCLBP after PLIF surgery. These conclusions, derived from a retrospective cohort, require validation in larger prospective studies that address limitations such as sample size constraints and potential model overfitting. The unique correlation of quadratus lumborum density (while its CSA was not predictive, likely due to measurement complexities inherent to its deep anatomical location) suggests that quality deterioration may independently contribute to pain. In clinical practice, quantitative assessment of core muscles using preoperative imaging lays the groundwork for identifying high-risk patients. Furthermore, our findings suggest that future research should explore the impact of intensive core muscle prehabilitation and postoperative neuromuscular functional rehabilitation as potential key strategies for preventing and managing rCLBP. Future research needs to delve deeper into the underlying mechanisms, dynamic evolution, and optimization of interventions, while also incorporating longitudinal assessment of muscle changes post-surgery to better understand causal relationships.

## Data Availability

The original contributions presented in the study are included in the article/Supplementary Material, further inquiries can be directed to the corresponding authors.
